# Technically sleeping? A clinical single-case study of a commercial sleep robot

**DOI:** 10.3389/fpsyg.2022.919023

**Published:** 2022-12-19

**Authors:** Siri Jakobsson Støre, Maria Tillfors, Erik Wästlund, Charlotte Angelhoff, Annika Norell Clarke

**Affiliations:** ^1^Department of Social and Psychological Studies, Karlstad University, Karlstad, Sweden; ^2^Crown Princess Victoria’s Child and Youth Hospital, and Department of Biomedical and Clinical Sciences, Linköping University, Linköping, Sweden

**Keywords:** arousal, hyperarousal, insomnia, sleep, wake after sleep onset

## Abstract

The Somnox sleep robot is promoted as sleep enhancing. The current study investigated individual effects, the acceptability and the safety of, and experiences with, a 3-week intervention in adults with insomnia. A repeated ABA single-case design (*n* = 4) was used to evaluate the effects of the sleep robot compared with baseline, as measured with a sleep diary and actigraphy. Pre-, post-, and 1-month follow-up assessments were conducted, measuring symptoms of insomnia, level of somatic arousal, and symptoms of depression and anxiety. Questions about adherence were included in the sleep diary. Individual interviews were conducted post intervention to explore the participants’ experiences with the sleep robot. The sleep diary and actigraphy data showed marginal differences, and if something, often a slight deterioration in the intervention phase. Three participants reported improvements regarding their sleep in the interviews compared with baseline, which mirrored the results on the questionnaires (insomnia and arousal) for two of the participants. The same three participants adhered to the intervention. Stable or improved self-assessed symptoms of depression and anxiety, and information from the individual interviews, suggest that the intervention is safe for adults with insomnia. The results regarding the effects of the sleep robot were mixed, and ought to be scrutinized in larger studies before confident recommendations can be made. However, the study supports the acceptability and safety of the intervention in adults with insomnia.

## Introduction

Insomnia is a common sleep disorder in adults, with a prevalence of 6–15% of the population in Western countries (e.g., Reynolds et al., 2019; Sivertsen et al., 2020). The criteria in the Diagnostic and Statistical Manual of Mental Disorders, Fifth Edition (DSM-5: American Psychiatric Association, 2013) for chronic insomnia (> 3 months) include difficulties falling asleep, staying asleep, or early morning awakenings, combined with decreased daytime functioning or significant distress. The symptoms have to occur three times a week for a period of 3 months for one to meet the criteria of chronic insomnia (American Psychiatric Association, 2013). Objective short sleep duration is not included in the diagnostic criteria, but has been found to be the most severe phenotype of insomnia (Vgontzas et al., 2013).

Models of insomnia can be grouped into physiological and cognitive models. Elevated physiological, cognitive, or emotional arousal (i.e., hyperarousal) is, however, included in most models, albeit playing different roles in different models. For instance, in the cognitive model of insomnia (Harvey, 2002), negative thoughts and worry lead to sleep-disturbing arousal. The hyperarousal model (Perlis et al., 1997; Riemann et al., 2010; Kay and Buysse, 2017) highlights arousal as a causal and maintaining factor in insomnia, with or without the involvement of negative thoughts. It has been highly debated whether insomnia is primarily caused by cognitive hyperarousal or physiological hyperarousal. As Harvey (2002, p. 886) writes, “Rather than being two opposing theories of insomnia, it is suggested that cognition and physiology should be viewed as co-operatively linked systems.” Hyperarousal has been found to be one of the most important pathophysiologic mechanisms in individuals with chronic insomnia (Vgontzas et al., 2013).

Cognitive-behavioral therapy (CBT-I) and pharmaceutical treatments are the gold standard treatments of insomnia (Riemann et al., 2017). CBT-I is an evidence-based, non-pharmacological treatment of chronic insomnia. It entails psychoeducational interventions (information about the connection between thoughts, feelings, behaviors, and sleep), cognitive interventions (attempts to change incorrect and/or dysfunctional thoughts about sleep), and behavioral interventions (sleep restriction, relaxation training, and more; Mitchell et al., 2012). Certain sleep medicines are associated with risks of addiction and adverse effects, and CBT-I has been found to have a higher long-time effectiveness in treating insomnia compared with sleep medication (Mitchell et al., 2012). Although CBT-I is the recommended first-line treatment, it is not always an available option due to factors such as a shortage of therapists with the right education (Soh et al., 2020). It is well established that relaxation has a beneficial effect on our physical and mental health (e.g., Stetter and Kupper, 2002; Manzoni et al., 2008). Relaxation is sometimes included in CBT-I (Cheung et al., 2018), but the effect of relaxation techniques alone on symptoms of insomnia is not well established (Baglioni, 2021, p. 292).

Several complementary and alternative methods (CAMs) have been suggested for insomnia, such as acupuncture, homeopathy, and yoga (Riemann et al., 2017). Though most alternative methods are not recommended due to insufficient empirical evidence regarding their efficacy and safety, engagement in them is common in people with sleep problems (Bertisch et al., 2012; Riemann et al., 2017). This is potentially a waste of time and money, and at worst, may cause unknown detrimental effects. Therefore, there is a need for independent testing of products that are advertised as sleep-promoting, e.g., sleep robots. A recent systematic review and network meta-analysis of the effects of companion robots on sleep in adults concluded that robot interventions did not have positive effects on sleep compared with plush toys and treatment as usual, but also that future studies should include a thorough screening of participants, and study robots especially made to target sleep (Støre et al., 2022).

**Figure 1:**
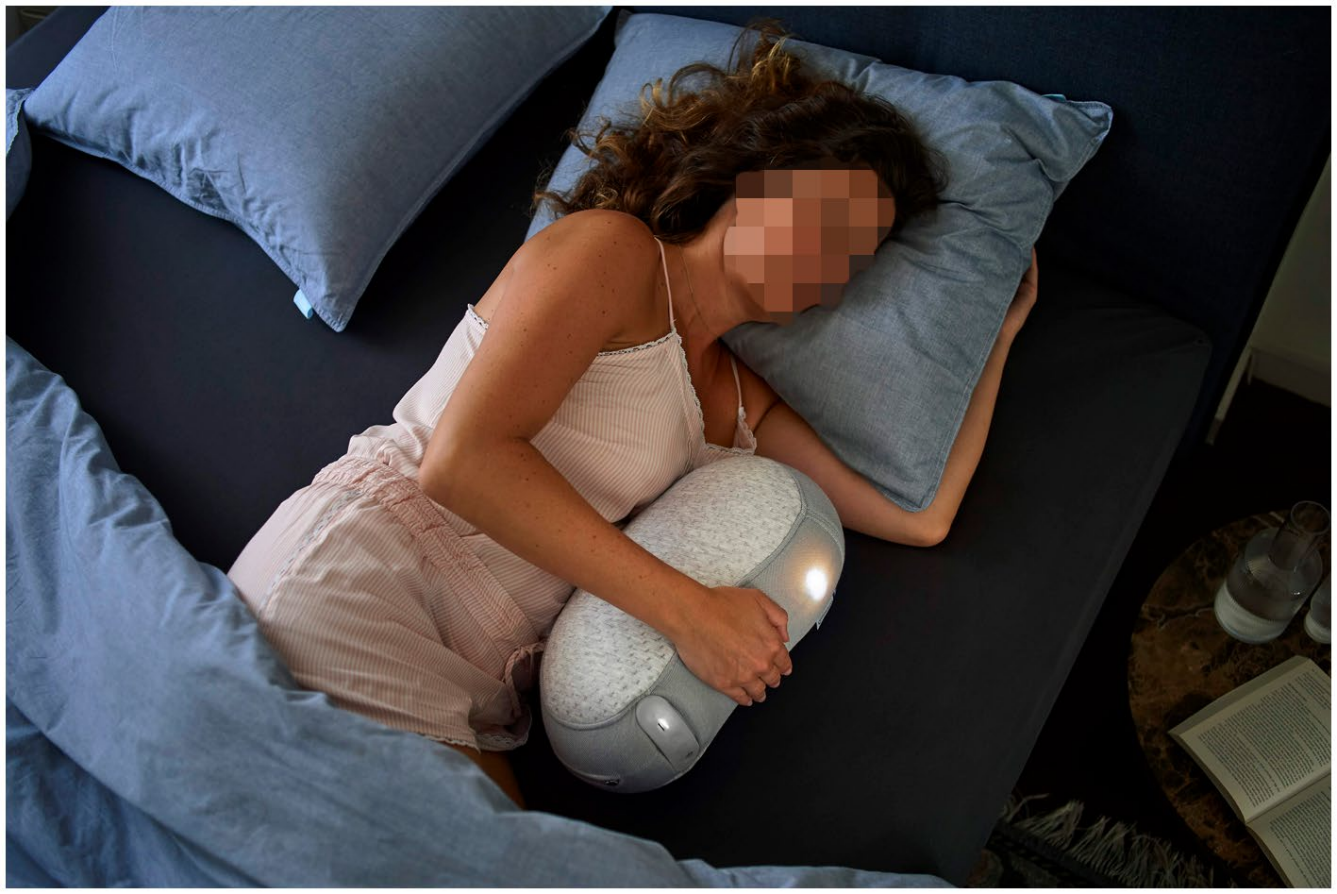
The Somnox sleep robot. Reproduced with permission from Somnox, available at https://somnox.com.

The Somnox^™^ sleep robot (see Figure 1) is a bean-shaped cushion designed to help people breathe more calmly and, as a result, fall asleep faster (Somnox, 2021). Slow breathing activates the parasympathetic nervous system, which stimulates relaxation (Russo et al., 2017). This gives us reason to believe that the robot, which supposedly promotes relaxation through slow and deep breathing, may have an effect on symptoms of insomnia and somatic arousal.

In recent years, robots have been developed to support people with everything from care tasks to social companionship (Silvera-Tawil et al., 2015; Fischinger et al., 2016). A robot is an artificial intelligence device that senses and acts with purpose to do something useful (Sætra, 2020). Previous research has found positive effects of certain robots on several psychological and physiological factors, such as loneliness and stress (Tasi et al., 2018; Meißner, 2020). Technology is developing at a rapid pace, and the research on technological products often lags behind (Meißner, 2020). Given that the results of studies such as the current one are likely to be used for commercial purposes, it is crucial to support people with insomnia through impartial research on products that claim to improve sleep, offering trustworthy information about the products’ effects and safety.

The aim of the current study was to evaluate whether a 3-week intervention with the sleep robot is effective, acceptable, and safe for adults with insomnia. The research questions were as follows:

- Does the sleep robot intervention have any effects on the participants’ symptoms of insomnia and sleep-disturbing arousal compared with baseline, as measured with a sleep diary, actigraphy, and questionnaires?
- Are the participants compliant in their use of the sleep robot, as measured with a sleep diary?
- Is the intervention safe for adults with insomnia, in that their mental health symptoms, as measured with a questionnaire of depression and anxiety symptoms, and daytime symptoms reported in the sleep diary, are stable over the treatment phase?
- How do the participants experience their use of the sleep robot, as disclosed in the individual interviews?

## Materials and methods

### Participants and procedures

Recruitment was conducted through the university’s webpage and through social media. Those who were interested in participating in the study were contacted by phone with more information. Those who were still interested went through a two-stage screening conducted by phone. In the first part of the screening, the participants were screened for insomnia using the Insomnia Severity Index (ISI: Bastien et al., 2001) and arousal using the Pre-Sleep Arousal Scale (PSAS: Nicassio et al., 1985). The first part of the screening considered symptoms experienced the last 7 days. Those who scored 11 or above on the ISI, indicating insomnia symptoms on a clinical level (in line with Bastien et al., 2001), and 10 or above on the somatic scale of the PSAS (in line with Jansson-Fröjmark and Norell-Clarke, 2012) went through to the second stage of the screening, which consisted of two structured clinical interviews, administered by the first author SJS (PsyD): (1) The Mini International Neuropsychiatric Interview (M.I.N.I.: Sheehan et al., 1998) and (2) the Duke Structured Interview for Sleeping Disorders (DSISD: Edinger et al., 2004). SJS had extensive clinical practice with the M.I.N.I. prior to the study, and received training on the DSISD from the experienced last author (ANC). These interviews were conducted to ensure that the participants did not meet the criteria of any other psychiatric or sleep disorder that offered a more plausible explanation of their current symptoms of insomnia. The screening lasted approximately 1 h 30 min altogether.

The first five people who showed interest in the study were recruited. Five was deemed to be sufficient considering the pilot nature of the study, the single-case research design, and due to the limited number of robots at hand combined with the fact that we wanted all participants to receive the intervention simultaneously as this was during the unstable times of the COVID-19 pandemic. One participant went through the first stage of the screening but did not complete it due to life circumstances. A number of demographic factors were collected during the screening: age, sex, marital status, employment, education, whether the participants were born in Sweden or not, and the number and age of children in the household (see Table 1). Age ranges are presented as opposed to specific ages in order to protect participant anonymity. Eligible participants were (1) Swedish-speaking (2) adults (18+) with (3) symptoms of insomnia on a clinical level, i.e., meeting the DSM-5 criteria, (4) with no other sleep disorder, or meeting the criteria for another sleep disorder but being adequately treated for it, and (5) with no medical or psychiatric diagnosis that could better explain the current symptoms of insomnia. Prescribed sleep medications were monitored and had to be stabilized on an optimal dose prior to the trial. There were no dropouts from the study post-screening. Ethical approval was obtained from the Swedish Ethical Review Authority (DNR 2020-06975) prior to the study.

**Table 1:** Demographic information.

Participant	Age span	Gender	Highest education	Employment	Marital status	Born in Sweden	Children in household	Sleep medication use
1	40–49	Female	University/college	Full-time	Separated/Single	Yes	Yes	No
2	50–59	Female	University/college	Full-time	Married	Yes	Yes	No
3	60–69	Male	High-school	Full-time	Married	Yes	Yes	No
4	60–69	Female	University/college	Full-time	Married	No	No	Yes

All participants met the DSM-5 criteria for insomnia. Wake after sleep onset was the most prominent insomnia symptom for all participants. Participants 2 and 3 estimated themselves to have suffered from insomnia for 5 years, whereas Participants 1 and 4 declared that they had had sleep problems of this magnitude for about 10 years. Three participants did not fulfill the criteria of any comorbid psychiatric, somatic, or sleep disorder besides insomnia. Participant 4 met the criteria for a major depressive episode in full remission. She still medicated with antidepressants, mainly for her insomnia, which she had suffered from prior to the depressive episode.

### Design

The current study used a repeated ABA single-case design. The baseline was 2 weeks long and entailed that the participants kept a daily sleep diary (standard recommendations are at least 3–5 baseline points). As Krasny-Pacini and Evans (2018, p. 171) write, “the more points in baseline, the more likely an intervention phase will be able to be differentiated from the baseline if an effect exist.” The baseline phase was followed by a 3-week intervention phase in which the participants continued to complete the sleep diary and an additional week of sleep diary post-intervention (“baseline”), meaning 6 weeks in total.

### Measures

The treatment was evaluated in multiple ways. Firstly, the participants kept a sleep diary (the Consensus Sleep Diary: Carney et al., 2012) on a daily basis for six consecutive weeks. The sleep diary included questions about sleep onset latency (SOL), wake after sleep onset (WASO), and total sleep time (TST), in minutes, among other things. Sleep efficiency (SE) was calculated by dividing TST by the total time in bed (in minutes) multiplied by 100 to convert it into percentages. Also included in the sleep diary were questions about whether, how, and for how long the participants had used the sleep robot during the intervention phase, as a measure of adherence, and questions about daytime symptoms (e.g., daytime alertness and irritability), as a measure of safety. Furthermore, the participants wore wrist actigraph units (Actigraph Link GT9X) for Weeks 2, 4, and 6 of the study. The actigraphy constituted objective assessments of the participants’ sleep by measuring their nighttime movements (Cole et al., 1992). The 3 weeks chosen, as opposed to collecting actigraphy data throughout the study, were due to matters of logistics (number of available actigraphs, the battery-life, etc.), but also to lessen the participation burden.

Furthermore, the participants filled out the following self-assessment forms pre- and post-intervention, in addition to a 1-month follow-up: (1) the Insomnia Severity Index (ISI), which is the first line evaluation of symptoms and treatment of insomnia; (2) the Pre-Sleep Arousal Scale (PSAS), which measures the level of physical and mental arousal (somatic scale); and (3) the Hospital Anxiety and Depression Scale (HADS: Zigmond and Snaith, 1983), which measures the level of symptoms of anxiety and depression. The ISI consists of seven items and has a score range of 0–28, where higher values indicate more severe self-assessed insomnia symptoms. The cut-off score of 11 was used in the current study to differentiate between clinical and non-clinical levels of symptoms. A change of −9.9 on ISI is considered a marked improvement, a change of −8.4 a moderate improvement, and a change of −4.7 a slight improvement (Morin et al., 2011). The PSAS consists of a mental and a somatic scale, where the somatic scale has eight items with a score range of 8–40. The cut-off score of 10 was used in the current study to indicate somatic hyperarousal. The HADS questionnaire, measuring anxiety and depression symptoms, has seven items measuring anxiety symptoms and seven items measuring depression symptoms for a total of 14 items. The HADS has a score range of 0–21, where higher scores indicate more symptoms of anxiety and depression. A score of 8 or more on either scale indicates a clinical level of the symptoms in question. The HADS constituted a measure of adverse effects and hence safety, similar to Quartly-Scott et al. (2020). After the intervention, the participants were interviewed individually about their experiences with the sleep robot. See the Supplementary material for the interview guide.

### Intervention

The Somnox sleep robot is a firm cushion whose sounds and movements imitate breathing. The “breathing” is thought to have a calming effect on the user and possibly reduce the time it takes to fall asleep. The participants were trained in how to use the robot prior to the intervention. This training took about 15–20 min. The participants went through a 3-week intervention of daily at-home use of the sleep robot. The sleep robot was fixed on a 30 min default program (“sleeping”) with a 1:2 ratio of inhalation and exhalation. The participants were asked to use the sleep robot every night in bed, holding the robot against their abdomen with the aim of falling asleep with it, and to use the robot after any unwanted awakenings. The participants were also asked to report in their sleep diaries how and for how long they had used the sleep robot.

### Analysis

WASO, SOL, TST, and SE were assessed with a sleep diary for six consecutive weeks, and with actigraphy for certain weeks of the study (weeks 2, 4, and 6; i.e., the last baseline week, the mid-intervention week, and the first week post-intervention). The data were visually analyzed. The medians of the sleep diary variables were calculated for all three phases of the study, for comparison. The median was chosen over the mean, as the former is more representative when the data distribution fluctuates, as for people with sleep problems. Furthermore, the percentages of all non-overlapping data (PAND) were calculated to compare the baseline and intervention phases of the sleep diary variables for each participant. The PAND is defined as “the smallest number of datapoints from either phase whose removal would eliminate all data overlap between two phases” (Parker and Vannest, 2009, p. 360). When interpreting PAND, 90% or above is considered a very effective treatment, 70–90% is considered an effective treatment, 50–70% is considered an uncertain effect, and 50% or less is considered an ineffective treatment (Parker et al., 2011). Nonoverlap methods go hand in hand with visual analysis and are more robust than means or medians with very skewed data (Parker et al., 2011). The explorative interviews were audio-recorded and transcribed at the semantic level. The current study focused on questions concerning how the participants experienced the sleep robot intervention (mainly questions 4–7 in the interview guide; see the Supplementary material).

## Results

**Figure 2:**
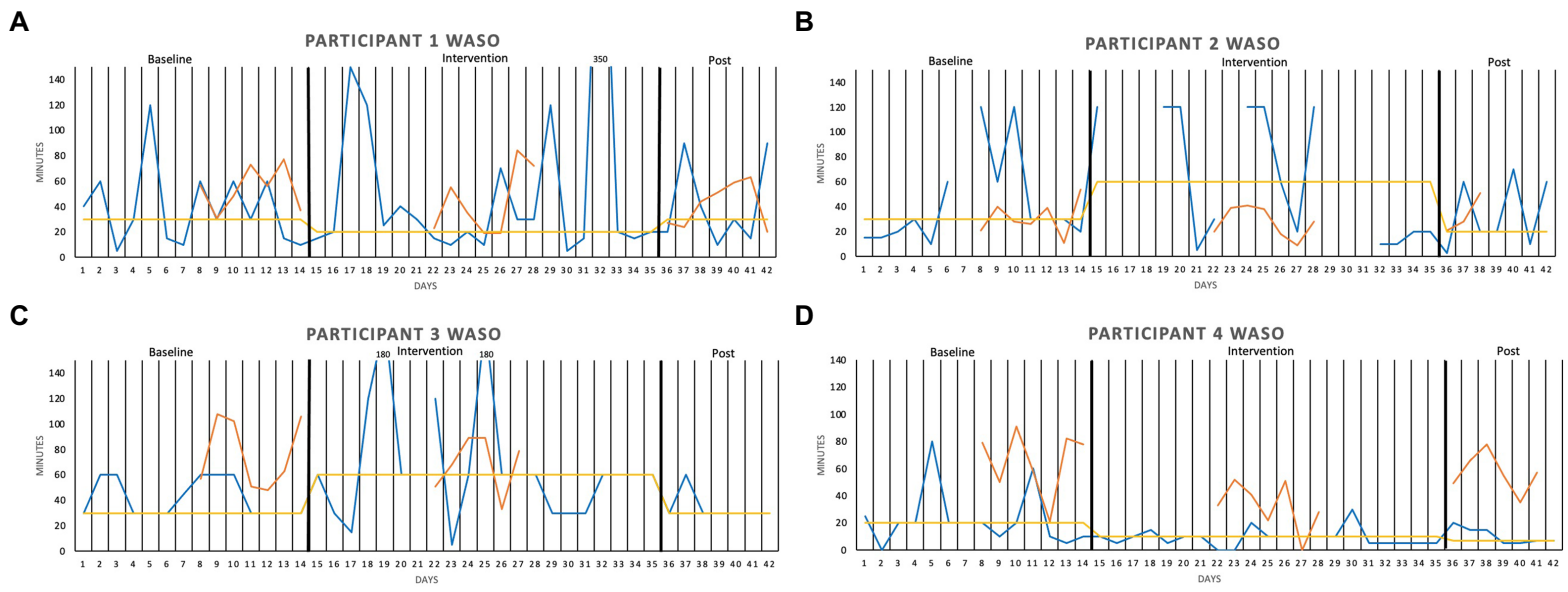
Wake After Sleep Onset (WASO, in minutes) measured over two baseline weeks, three intervention weeks, and 1 week post-intervention, for **(A)** Participant 1, **(B)** Participant 2, **(C)** Participant 3, and **(D)** Participant 4. The sleep diary is represented by the blue lines, the actigraphy by the orange lines, and the median of each phase by the yellow lines.

Wake after sleep onset (WASO), which is graphically depicted in Figure 2, was visually inspected. Of the daily measures, we focused on WASO, as this was the most salient insomnia symptom at baseline for all four participants. The sleep diary data fluctuated in all three phases of the study for all participants, making it difficult to judge whether any changes had occurred by visual inspection only. The same was true for the actigraphy data. There were also discrepancies between the sleep diary and the actigraphy in a nonsystematic way, complicating the interpretation of data even further.

When adding median lines of the sleep diary data in all three phases of the study, the intervention seemed to have had a somewhat favorable effect on Participants 1 and 4, and a slight unfavorable effect on Participants 2 and 3, compared with baseline. For Participant 1, the median WASO was 30 min in the baseline phase, 20 min during the intervention, and 30 min post intervention. For Participant 2, the median was 30 min in the baseline phase, 60 min during the intervention, and 20 min post intervention. The same pattern can be seen in Participant 3’s graph (30, 60, 30 min, respectively), except that the baseline and the post-intervention medians are the same. Participant 4 had, on the other hand, a lower median in the intervention phase (10 min) compared with the baseline (20 min), and the slight favorable trend continued into the post-intervention phase of the study (7 min).

Equivalent graphs of sleep onset latency (SOL), total sleep time (TST), and sleep efficiency (SE) can be found in the Supplementary material. SOL increased somewhat in the intervention phase for Participants 1 and 2, remained the same for Participant 3, and decreased for Participant 4, compared with baseline. Participants 1, 2, and 4 all had slightly higher TST medians during the intervention phase, while Participant 3 had somewhat less TST during the intervention, compared with baseline. Regarding SE, it seemed to remain basically the same in all three phases of the study for Participant 1. For Participants 2 and 3, the sleep was slightly less efficient in the intervention phase, compared with baseline, whereas Participant 4 seemed to have experienced a slight favorable effect in this regard (i.e., the sleep efficiency was higher during the intervention compared with baseline, and the favorable trend continued into the post-intervention phase).

To validate what might visually appear to be favorable or unfavorable effects of the sleep robot, and to check that we did not miss any effects, PAND was calculated for all the sleep diary variables (WASO, SOL, TST, and SE) of all the participants (see Table 2). Here, one wants to see scores as close to 100 percent as possible, but all the effects of the sleep robot on the sleep diary variables were small to non-existent.

**Table 2:** The percentage of all non-overlapping data (PAND) for wake after sleep onset (WASO), sleep onset latency (SOL), total sleep time (TST) and sleep efficiency (SE).

	Participant 1	Participant 2	Participant 3	Participant 4
WASO	0	3.6	17.1	0
SOL	2.9	18.2	11.4	2.9
SE	5.7	10.7	2.9	17.1
TST	5.7	17.9	14.7	5.7

**Figure 3:**
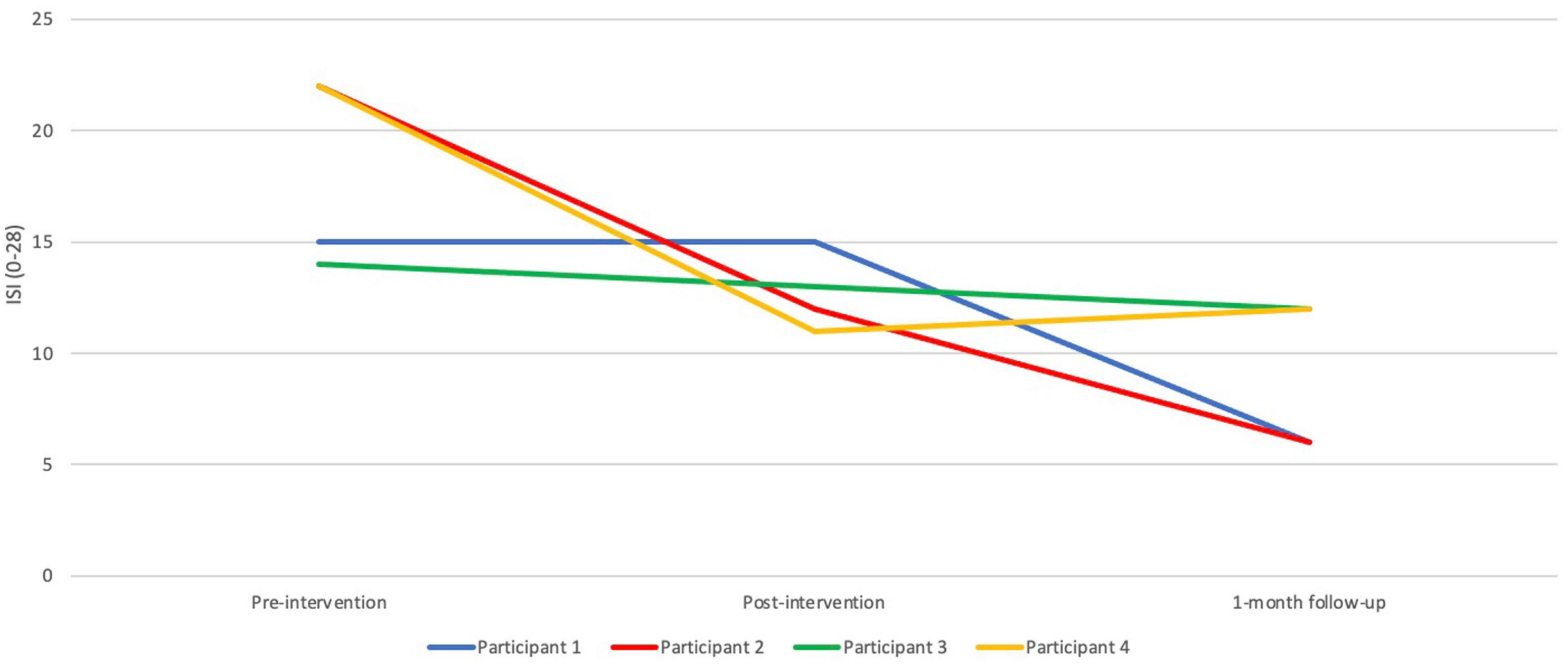
Pre-, post-, and 1-month follow-up assessments with the Insomnia Severity Index (ISI).

For Participants 2 and 4, there were relative reductions on the Insomnia Severity Index (ISI) from pre- to post-intervention, which remained at the same level for Participant 4 at the 1-month follow-up. For Participant 2, there was a further reduction at the follow-up, to an ISI score that was actually below the cut-off score, indicating that the participant no longer met the criteria for insomnia. This was also true for Participant 1, who had the same ISI score pre- and post-intervention, but a reduction after the intervention at the follow-up assessment. Participant 3 had a trivial reduction from pre- to post-, and further to the follow-up assessment. Figure 3 depicts the ISI results graphically.

**Figure 4:**
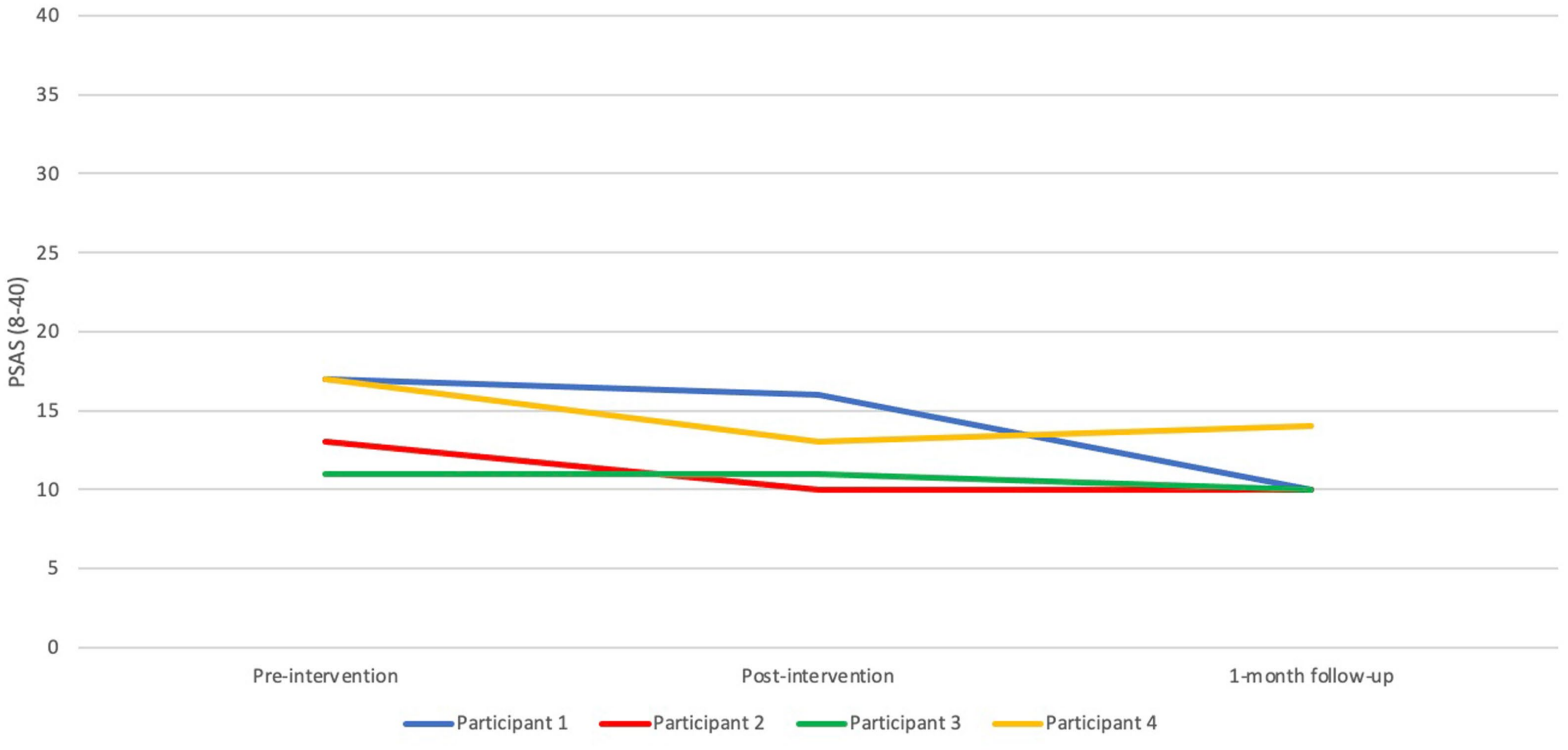
Pre-, post-, and 1-month follow-up assessments with the Pre-sleep arousal scale (PSAS).

Regarding the Pre-Sleep Arousal Scale (PSAS), there were slight reductions from pre- to post-intervention for Participants 1, 2, and 4, with a larger reduction for Participant 1 from the post-assessment to the follow-up assessment. The favorable effect on the PSAS remained at the same level for Participant 2 at the 1-month follow-up, whereas a slight unfavorable change had occurred for Participant 4 at the follow-up. Participant 3 scored the same at the pre- and post-assessments, with a slightly lower score at the follow-up. None of the participants had a PSAS score below the cut-off of 10, either post-intervention or at the follow-up. Figure 4 graphically depicts the PSAS results.

### Acceptability

In the current study, acceptability of the intervention was operationalized by the level of treatment adherence as measured by a sleep diary. Participant 1 used the robot for 15–120 min every night (mean 35.26, SD 27.31), as stated in the sleep diary. Participant 2 reported to have used the sleep robot for 15 out of 21 nights, between 30 and 180 min (mean 85.38, SD 34.31). Participant 3 only used the robot for 4 out of 21 nights, i.e., very low adherence—and did not report for how long the robot was actively used those four nights. Participant 4 reported to have used the sleep robot every night of the study between 90 and 120 min (mean 120.00, SD 26.83). To sum up, three out of four participants had a high level of treatment adherence, whereas one had low adherence.

### Safety

**Figure 5:**
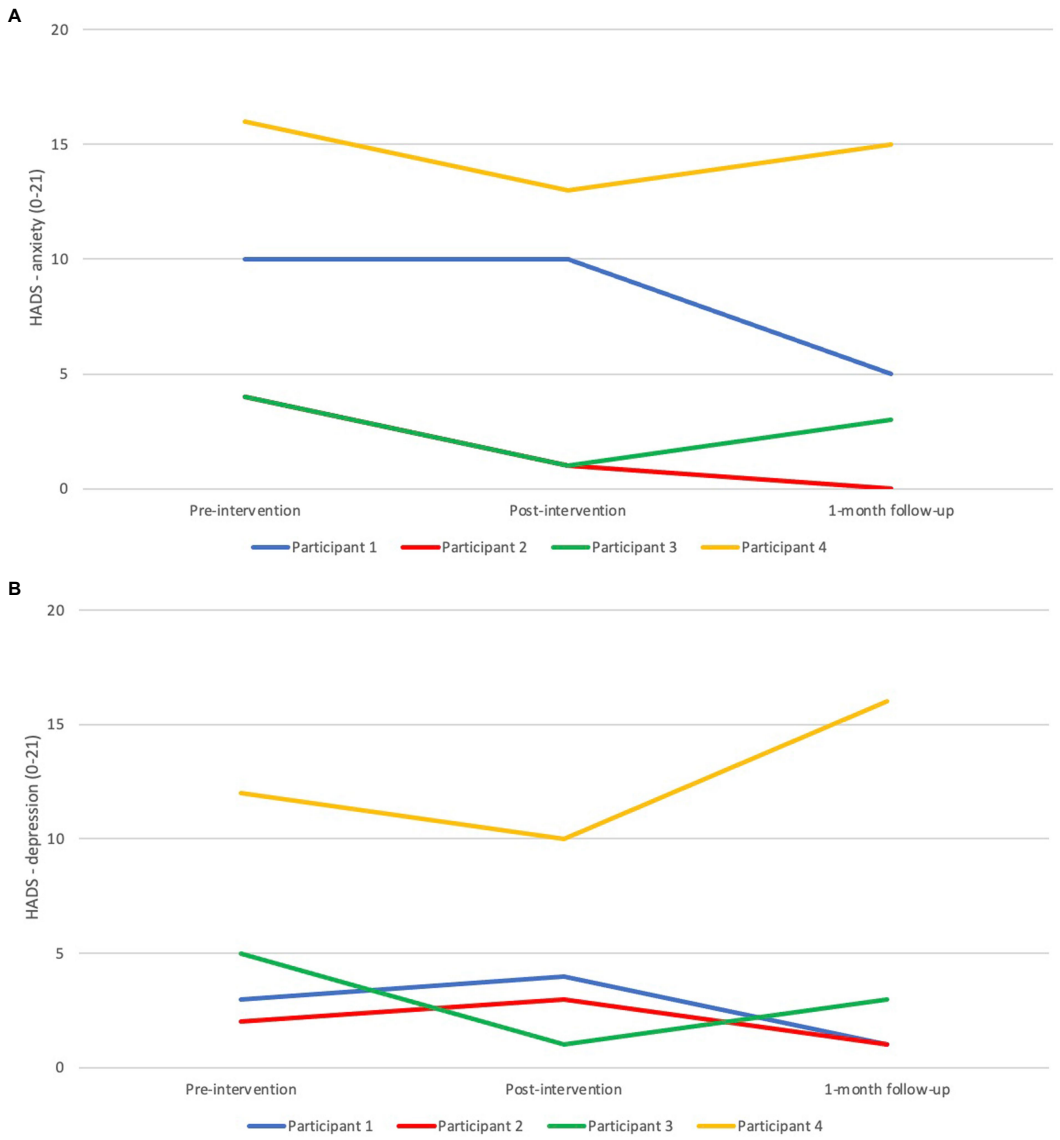
Pre-, post-, and 1-month follow-up assessments with **(A)** the Hospital anxiety and depression scale (HADS)—anxiety scale, and **(B)** the HADS—depression scale.

In the current study, safety of the intervention was operationalized as the absence of adverse effects as assessed by the HADS (anxiety and depression) and the sleep diary (daytime fatigue). In Figure 5, one can see that Participant 1’s anxiety score remained the same from pre- to post- assessment, but that the score was halved by the 1-month follow-up, ending up at a nonclinical level post intervention. The depression scores varied slightly between the three assessment points. Regarding Participant 2, anxiety was reduced from the pre- to the post-assessment, with a further reduction at the follow-up assessment. The depression score varied slightly for the pre-, post-, and follow-up assessments. For Participant 3, the anxiety and depression scores were at their lowest immediately after the intervention. The same pattern was true for Participant 4, albeit with higher levels of both anxiety and depression, above the cut-offs at all measurement points. None of the participants filled out the questions about daytime fatigue in the sleep diary in a strict way (i.e., many items were missing), why it was difficult to assess any changes quantitatively, but the participants did not seem to report more daytime fatigue during the intervention phase compared with the baseline or the post-intervention phases. To sum up, the participants generally did not report higher levels of anxiety and depression post-intervention, and they also did not seem to experience more daytime fatigue during the intervention phase compared with the other phases of the study.

### Experience

After the intervention, the participants were interviewed individually by the first author (SJS). The interviews lasted 7.23–29.47 min. An interview schedule with open-ended questions was prepared beforehand. The interview schedule (see the Supplementary material) was adhered to, albeit flexibly, to follow up on what the participants disclosed. In the interviews, we focused on how the participants experienced the sleep robot and the intervention.

Participant 1 described how it took some time to get used to the robot and how she experienced a gradual positive change in her sleep between the second and the third week of the intervention. She found it soothing to focus on the robot’s breathing. She described waking up as many times as before during the nights of the intervention period but falling asleep faster with the robot. She reported going from being awake a couple of hours most nights to being able to fall back asleep in just 20 min with the robot, which she experienced as a “pretty big difference,” albeit not a life-changing one. She would have wanted a longer intervention period and would even have liked to keep the sleep robot:

P1: Now I wonder how I’ll go on without “Rob” [laughter]. No, but it will be interesting to see how it goes this week, as it’s the first week without the robot. To see if I’m able to fall asleep, or if I’m able to visualize the robot or something in any way, and see if it sort of continues to have an effect.

Participant 2 also needed some time to get used to the robot, which she did during the third week of the intervention. She described that the robot “forced” her to relax by having her breathe to match the robot’s slower pace. This felt a bit uncomfortable early in the sessions, when she was still worked up, but eventually resulted in relaxation and better sleep. She described the robot as a reminder of relaxation and sleep and the conditioning effect she felt when she pressed the power button. She further described that while using the robot, she never engaged in sleep-delaying behaviors such as scrolling through feeds on her smartphone, which she assumed contributed to the favorable effects. She described feelings of happiness resulting from better sleep due to the intervention and stated that other people in her life had noticed a difference in her as well:

P2: My husband said it as well. Yeah, but I woke up and felt like, even when I woke up in the middle of the night, I was able to fall asleep again, so when I woke up, I was sort of… happy. Otherwise, I’m just like, no one wants to talk to me [laughter]. Uh, so I, there’s a difference, there’s a difference. I feel rested.

Participant 3, who did not adhere to the intervention, suffered from middle-of-the-night awakenings and did not want to use the robot then for the fear of getting less sleep. He had also expected things other than what the robot provided:

P3: I feel like I could really benefit from a robot-like object that measures my sleep during the night. To see how much, to see how much deep sleep I get. And my heart frequency, and so on. That kind of knowledge would benefit me more than the procedure of falling asleep.

Participant 4 described her experience with the robot as “love at first sight”—an immediate positive effect that she did not anticipate. She experienced a softer transition from wakefulness to sleep with the robot:

I: Could you tell me a bit more about that, that you “disappear” with the robot?

P4: Yes, I disappear into nothingness. I mean, I don’t know what I, I don’t notice that I fall asleep. Before, I used to think a lot about being about to fall asleep, very self-consciously, but with the robot, there was no such boundary between being awake and falling asleep. The transition was so mild. And comfortable, so I didn’t notice it. And that is probably the best and most natural way, really. The way it should be, but I hadn’t experienced it before.

The participant continued to describe how the robot distracted her from her usual ruminating thoughts and how she had tried every means of distraction before, such as television and books but the sleep robot being the most effective one.

All participants reported the documentation of their sleep in the sleep diary as an important part of the intervention and a contributing factor to the favorable effects that three of them described it to have had. All participants thought that the buttons on the sleep robot could be optimized. Participant 2 reported that the on/off button affected how much she used the robot after unwanted awakenings, as she did not want to turn on the light and become even more awake. Participants 1 and 2 spontaneously commented on the shape of the robot, describing the robot as harder and more awkward than they had anticipated.

## Discussion

The current study is an independent evaluation of individual effects, the acceptability and the safety of, and experience with, the Somnox sleep robot in adults with insomnia. Our focus was on wake after sleep onset (WASO), as this was what the participants reported to be their main sleep problem. WASO, as measured with both a sleep diary and actigraphy and visually inspected, fluctuated highly in all three phases of the study for all four participants. When adding median lines in the different phases of the study of the sleep diary data, there seemed to be no change for Participant 1, a slight worsening in the intervention phase for Participants 2 and 3, and a minimal improvement for Participant 4, when comparing the intervention phase to the other phases. However, the PANDs showed that all scores were well below the 50% limit for an uncertain effect of the intervention. The same was true for sleep onset latency (SOL), total sleep time (TST) and sleep efficiency (SE). This is partly in line with previous research on other insomnia treatments, which shows for small (if any) changes in TST and SE (Trauer et al., 2015). As SOL was not the most salient insomnia symptom for any of the participants, the small effects on SOL make sense. We were, however, surprised by the small effect of the intervention on WASO, the participants’ primary symptom.

The favorable effects described in the interviews reflected the self-reports of insomnia severity for two of the participants. Participant 2’s ISI score was reduced from 22 to 12 from pre- to post-intervention, whereas Participant 4’s score changed from 22 to 11, both representing marked improvements (Morin et al., 2011). The same two participants reported relatively larger reductions on the PSAS from pre- to post-intervention, in support of the connection between hyperarousal and insomnia. There were discrepancies between the sleep diary and the results on the ISI, which may be explained by the fact that the first measures insomnia symptoms on a daily basis, inevitably affected by recent events, whereas the ISI reflects more general insomnia symptoms over a week rather than estimated sleep times. The ISI is also considered to measure qualitative experiences of one’s sleep. The fact that the participants liked the robot but did not show any dramatic changes in the sleep diary could reflect that relaxation in itself is pleasant and has been found to have positive effects on our bodies and our minds. Regarding the acceptability of the intervention, three of the four participants had a high level of adherence. No adverse effects were detected in the self-reported symptoms of anxiety and depression post-intervention, or regarding daytime fatigue or in the interviews, which speaks to the safety of the intervention.

One strength of the current study is that the robot used in the current study has been produced to target sleep, as opposed to previous studies on robots’ effects on sleep (Støre et al., 2022). Another strength is the thorough screening process of the participants. Other strengths include the use of outcome measures with sound psychometric properties and the use of both subjective and objective sleep measures. There were, however, discrepancies between the two, complicating the interpretation of data. People with insomnia have been found to have more variable sleep at home compared with in a laboratory, perhaps due to the social situation, keeping the participants from staying in bed for as short or long a time as they would at home (Hauri, 1989). Hence, the current study can be said to have high ecological validity, which is a strength. We could in no way control how the participants used the sleep robot, but the fact that we collected self-reported data on adherence is yet another strength of our study.

The study also has some limitations. Firstly, the large fluctuations in all phases for all the participants lower the study’s internal validity, especially since the fluctuations did not stabilize at baseline. Visual inspection is affected by subjectivity and inconsistency, and even experts within the same fields have been said to often disagree about certain results and the reliability of those results (Kazdin, 2003, p. 213). Visual inspection also demands a greater effect for it to be a clear change visually, compared with tests of statistical significance, which makes it difficult to judge whether the improvement is due to the intervention or to a natural development over time (Kazdin, 2003, p. 214). This was certainly the case in our study - and the reason that we calculated the effect sizes through the percentage of all non-overlapping data (PAND), adding to the strengths of our study. Another important limitation is the missing sleep diary data that may have affected the median values provided, and the fact that we only collected actigraphy data certain weeks of the study. Our results regarding the effects of the Somnox sleep robot cannot be generalized due to the small number of participants. The small scale is, however, reasonable for a first study on such a novel intervention before conducting larger studies (Kazdin, 2003, p. 220). All four participants spontaneously stated that they thought keeping a sleep diary had a positive effect on their sleep. Sleep diaries are, in fact, an essential part of CBT-I for insomnia (Riemann et al., 2017). It is therefore important to control for the effect of the sleep diary in a randomized controlled trial.

## Conclusion

The results regarding the effects of the sleep robot were mixed, and ought to be scrutinized in larger studies before confident recommendations can be made. For now, the insufficient empirical evidence implies that the Somnox sleep robot should not be recommended. However, people with insomnia, and researchers conducting future larger studies on the Somnox robot, can rely on the current study for an independent evaluation in support of its acceptability and safety.

## Data availability statement

The raw data supporting the conclusions of this article will be made available by the authors, without undue reservation.

## Ethics statement

The studies involving human participants were reviewed and approved by Swedish Ethical Review Authority. The patients/participants provided their written informed consent to participate in this study. Written informed consent was obtained from the individual(s) for the publication of any identifiable images or data included in this article.

## Author contributions

ANC, MT, and SJS designed the study. EW provided the sleep robots. SJS collected and analyzed the data and drafted the manuscript. CA provided the actigraphy equipment and analyzed the data from the actigraphs. All authors contributed to the article and approved the submitted version.

## Conflict of interest

The authors declare that the research was conducted in the absence of any commercial or financial relationships that could be construed as a potential conflict of interest.

## Publisher’s note

All claims expressed in this article are solely those of the authors and do not necessarily represent those of their affiliated organizations, or those of the publisher, the editors and the reviewers. Any product that may be evaluated in this article, or claim that may be made by its manufacturer, is not guaranteed or endorsed by the publisher.
